# A Retrospective Clinical Audit of Bracket Failure among Patients Undergoing Orthodontic Therapy

**DOI:** 10.1155/2020/8810964

**Published:** 2020-12-15

**Authors:** Dashrath Kafle, Rajeev Kumar Mishra, Md Riasat Hasan, Takashi Saito

**Affiliations:** ^1^Department of Orthodontics, Kathmandu University School of Medical Sciences, Dhulikhel Hospital, Dhulikhel 45200, Nepal; ^2^Division of Clinical Cariology and Endodontology, Department of Oral Rehabilitation, School of Dentistry,Health Sciences, University of Hokkaido, Hokkaido 061-0293, Japan

## Abstract

**Objective:**

Bonding failure is a problem in orthodontic therapy and has been associated with orthodontic emergencies and delayed treatment time. The aim of this study is to determine the bracket failure rate among patients undergoing orthodontic therapy.

**Materials and Methods:**

From the archives of posttreatment records, 200 total cases were selected, out of which 122 cases had detailed treatment records. Cases with incomplete records, large restorations, or enamel aberrations were excluded from the study. Descriptive statistics were applied to obtain sample characteristics, and a chi-square test was applied to compare the bracket failure between different categories.

**Result:**

Out of the 122 samples enrolled in the study, female and male patients comprised 71% and 29%, respectively. Class I malocclusions were the most common problem (56%), followed by Class II (41%) and Class III (3%). The bracket failure rate was 3.43%, and 32% of the patients had an incidence of bracket failure. There was no significant difference in bracket failure among genders (*p*=0.344), malocclusion types (*p*=0.191), or operators (*p*=0.188). The maxillary left quadrant was the most common site of bracket failure, followed by the mandibular right quadrant.

**Conclusion:**

Bracket failure is relatively uncommon. It is not affected by the gender or age of the patient or by malocclusion type. The maxillary left and mandibular right quadrants are the most common sites of bracket failure.

## 1. Introduction

The global prevalence of malocclusion is reported to be approximately 50–80% [1–3]. A number of studies on the Nepalese population also found similar results [4, 5]. However, malocclusions themselves comprise a vast area of research. Broadly, malocclusions are divided into Classes I, II, and III. Among these, Class I malocclusions are the most common type in the world, followed by Class II and Class III [1, 2, 4–8]. Not all malocclusions need orthodontic treatment. The need for orthodontic therapy is determined by the malocclusion's severity, its effect on the stomatognathic system, and the demands of the patient.

Orthodontic therapy consists of treating various malocclusions with different types of appliances. In contemporary orthodontic practice, preadjusted edge-wise systems are the most commonly used across the world [9]. In this system, brackets are bonded to the maxillary and mandibular teeth up to the second premolars. Orthodontic brackets are then adhered to on either the labial or lingual tooth surfaces and act as a medium for the delivery of forces. Before the invention of composite resin and acid etching techniques, brackets were welded on a band, and each tooth was banded. This was a time-consuming process and uncomfortable for patients. Banding was replaced after the development of composite resin and different generations of bonding agents. Although there is currently a very low rate of banding, bracket failure is one of the main factors for repeated emergencies and delayed treatment in orthodontics [10]. The terminal teeth, the molars, are either banded or bonded. In several studies, sufficient bonding strength of the molar tubes was achieved on the first and second molars, and the results were satisfying [11–13]. Thus, the bonding of molars is becoming increasingly more popular, as bonding requires less chair-side time, the bonding tubes are gingiva-friendly, and the interdental area is free of bands, which is more comfortable for patients. Molar banding is associated with the pain that results from the separator as well as the encroachment of the interdental area by the band material [14]. Moreover, banding is associated with the occurrence of dental caries and/or white-spot lesions [14–16].

Notably, however, bonding is a sensitive procedure. There are a number of steps involved during the bonding of attachments on the tooth surface. Overall, bonding failure was found to be as low as 0.6% and as high as 28% [10, 17–23]. In general, a bracket failure rate below 10% has been suggested to be clinically acceptable. According to a study done by Stasinopoulos et al., the most common type of bracket failure occurs on the mandibular lower second premolar [10]. However, there are occasions where bracket failure occurs in other teeth, as well. The reasons for bracket failure might be associated with operator factors, patient factors, and/or material factors. With the development of newer adhesive resins, the prevalence of bonding failure is decreasing. The posterior teeth are more prone to bracket failure because of their location, the difficulty of moisture control during bonding, and the impact of masticatory force [24].

As bracket failure can be multifactorial, a study of Nepalese orthodontic patients is warranted, as such patients might be different than other populations due to the food habits and musculoskeletal patterns of the population [25], as well as the protocols followed by Nepalese clinicians. The aim of this study is to determine the failure rate of orthodontic brackets and compare this failure rate with other factors in patients presenting for orthodontic treatment at the Kathmandu University School of Medical Science (KUSMS).

## 2. Materials and Methods

This retrospective study was carried out at the Department of Orthodontics, Dhulikhel Hospital, Kathmandu University Teaching Hospital, Dhulikhel, Kavre, after approval (IRC no. 112/19) from the Institutional Review Committee of KUSMS. The orthodontic records of 200 debonded cases were selected from the archive of the orthodontic department. Based on the inclusion and exclusion criteria, only 122 cases were shortlisted and further analysed for study purposes. Only cases with treatment in both jaws and complete details of the treatment were included in this study. All the cases were treated by using 0.22” slot bracket (Mini Diamond, Ormco, USA). Bite turbos were used to disocclude the posterior teeth during initial alignment. Curve and reverse curve of Spee wires were used to level the overbite. Records with insufficient details, sectional and retreatment cases, and patients with heavy restorations, hypoplastic enamel, and/or craniofacial anomalies were excluded from the study. Data were collected in an Excel spreadsheet, which was later transferred to SPSS (version 20, IBM, New York, USA). Descriptive statistics were applied to determine the prevalence of bracket failure, and chi-square test was applied to determine the differences in the attachment failure between genders, malocclusion types, and operators. As there were many fewer samples in Class III category, further statistical analyses were not done on this category.

## 3. Results

Out of the 122 selected patients, 50 subjects had undergone extraction therapy in both jaws. Thus, 2240 total teeth were bonded with brackets for orthodontic therapy. Together, 39 patients and 75 brackets experienced failure, which represents 32% of the patients and 3.3% of the total brackets (Table 1). The most prevalent malocclusion was Class I, followed by Class II and Class III. Most patients were female (71.3%). The mean age of the patients was 19.9 years (19.9 ± 4.64). The most common site of bracket failure was the maxillary left side, followed by the mandibular right (Table 1).

There was no statistically significant difference on bracket failure rates among different malocclusion types (*p* = 0.191, Table 2). Likewise, the bracket failure rates between the two different operators and age groups of patients were shown not to be significant (*p* = 0.188, 0.184, Tables 3 and 4).

## 4. Discussion

This retrospective study highlighted the prevalence of bonding failure among patients attending a university dental clinic. The overall bonding failure was found to be 3.34%, and 32% of patients had experienced bracket failure during their treatment period. Similar studies done in other populations have found varying rates of bonding failure (0.6–27%) [21–23, 26, 27].

A study from Truk et al. found more minimal bonding failure with the total etching technique than under the use of a self-etching primer [23]. The authors found that there was no difference in the bonding failure rate between males and females, which is consistent with our study. In a very well-controlled study done over the period of 12 months, Jung MH found that bracket failure is more common in posterior teeth than in anterior teeth [28]. Jung's study found that there is a greater chance of bracket failure among young patients than older patients, which is inconsistent with our findings. In our study, both adolescents and adults had similar rates of bonding failure. In our study, the most common malocclusion was Class I, followed by Class II, which is consistent with the prevalence of malocclusions in the general population, as well as among orthodontic patients [1, 3, 4, 29, 30]. In our study, most of the subjects were female, which is again similar to numerous other studies done both nationally and internationally [5, 31, 32]. Globally, there are more female patients seeking orthodontic therapy than males. Our study found that the maxillary left quadrant is the most common site of bracket failure, which is different than the results of other studies [28]. This might be because the center used for our study performs the bonding of the maxillary arch first, followed by mandibular arch bonding after 1-2 months. Most bonding failure tends to occur within a few months of orthodontic therapy. Patients slowly become adapted to their brackets and tubes over time, and the type of malocclusion did not have any effect on the rate of bonding failure. Our findings are in agreement with those of a study by Millett et al. [33], which also found that the age and gender of the patient do not have any role in bonding failure. Furthermore, our study revealed that bracket failure is not significant between two operators, which is similar to the study of Millett et al. [33]. However, in this previous study, the bracket failure rate was recorded as 6%, which is higher than that found in our study.

## 5. Conclusions

There is no difference in the bracket failure rates among genders, malocclusion traits, and operators. The maxillary left and mandibular right quadrants are the most common sites of bracket failure.

## Figures and Tables

**Table 1:** Sample characteristics.

	Category	Number	%
Total patients		122	
Patients with bond failure		39	32

Patients by gender	Male	35	28.7
	Female	87	71.3

Distribution by malocclusion type	Class I	68	55.7
	Class II	50	41
	Class III	4	3.3
Mean age		19.9 ± 4.64	

Distribution of malocclusion by age	Below 12	2	
	12–16	25	
	17–20	43	
	Above 20	52	

Distribution of malocclusion by gender			

Class I	Male	17	
	Female	51	

Class II	Male	15	
	Female	35	

Class III	Male	3	
	Female	1	

Number of teeth	Before starting treatment	2440	
	Therapeutic extraction	200	

Number of brackets		2240	
Number of bracket failures		75	3.34

Patients by operator	Operator 1	57	
	Operator 2	65	

Site of failure	Maxillary right	6	17.6%
	Maxillary left	13	38.2%
	Mandibular left	5	14.7%
	Mandibular right	10	29.4%

**Table 2:** Comparison of bond failure by malocclusion type.

		Class I	Class II	*p*
Malocclusion	Failure	24	44	0.191
	Nonfailure	13	37	

**Table 3:** Comparison of bond failure among operators.

	Operator 1	Operator 2	*p*
Failure	21	18	0.188
Nonfailure	36	47	

**Table 4:** Comparison of bond failure among adolescents and adults.

	Adolescent	Adult	*p*
Failure	22	17	0.184
Nonfailure	38	45	

## Data Availability

The data used to support the findings of this study are included within the article.
